# "All Sorts and Conditions" of Women

**Published:** 1889-01-05

**Authors:** 


### The Doctor's Pulpit.

### "ALL SORTS AND CONDITIONS" OF WOMEN.

### I.—Preliminary Considerations.

"The science of woman" is one of the most progressive of the sciences: at the present moment much more progressive than the "science of man," of which it is a branch. The modern woman is decidedly interesting; she is so very human under all the wonderful variety of modern conditions. A man may cease to be a man and become, under pressure of environment, a mere doctor, or mathematician, or member of Parliament, or "mummy." But whatever a woman grows to, whether scholar, saint, or single-skirted fox-hunter she never ceases to be a woman. She still retains her eyes and her tongue; she can still be venomous when occasion serves; she can still give up body, soul, and spirit in complete self-abandonment for child, for man, for God. "Woman is the lesser man," says the Laureate. We cannot, offhand, endorse the proposition. She is the "other" man if you will; but before she can be pronounced the "lesser" there must be a good many definitions of greatness and smallness, and large inductions and comparisons. To man woman is the supremest object of interest among all the studies and businesses of his life. The boy is indifferent; the young man awakens to a new existence in her presence; the old man cannot die in perfect peace if she be not near.

Women are putting away the marks and badges of inferiority, and ranging themselves in life's race by the side of men. It is curious, instructive, and amusing to observe how men bear themselves under the new competition. It is a kind of test in mental chemistry which shows in a new way what many men are really made of Some of them scowl, some snort, some affect indifference, some contempt, some even profess to smile. But women retain their gravity and their position under the trying circumstances. They have come to the battle, like David from the sheep pastures, and it is clear that like him they intend to stay and to fight with such strength and such weapons as they have.

Well, but let us look at the facts "without prejudice," as the lawyers say. To outward appearance women belong to the same class of animals as men. When their brains are examined it is impossible to discover the difference. No doubt the average woman has a rather smaller head than the average man, and therefore her brain will usually be smaller. But the general outline and the subdivision and structure of the organ are in the two sexes so similar as to be practically identical. So much is this the case that if two brains of about the same size were examined side by side it would be impossible for the most accomplished scientist to say whether one was a man's and the other a woman's, or whether both were men's or both women's. That woman is different from man in many respects is admitted; but "difference" does not necessarily constitute "inferiority." If it be said that the intellect and character of woman are the "complement" of the intellect and character of man, the statement may with equal propriety be put the other way about, and man may be declared to be the "complement of woman." Many men, and women too, of course, have such poor mental powers that they are not conscious of the deficiencies that characterise the human intellect. The average man is not the typically complete man. The average man is a kind of "part" man—intellectually and morally. He has some great mental powers; but all his mental powers are not great. He has some strong moral faculties, but all his moral faculties are not equally strong. He may be an orator but not a poet; a musician but not a mathematician; a scientist but not a man of business; a man, but not also a woman. Now, a typically complete man would possess all the mental powers, and all the moral faculties, and each one of them in its highest perfection. He would be orator, poet, musician, scientist; he would be man and woman too. These elementary truths are well understood, though not always remembered in practice. They are expressed by such common sayings as these: "No man has all the talents"—"Every man to his trade"—"It takes all sorts to make a world"—"You cannot make a silk purse out of a sow's ear."

But the ardent evolutionist, especially if he be at the same time an equally ardent believer in the sublime religious ideas which have found their way into the world, and effected a lodgement that happily promises to be permanent—the man of this type of mind sees the possibility of a perfected humanity, and regards the conversion of that possibility into an actuality as the greatest and worthiest work to which he can put his hand. Such a man, recognising with clear conviction those distinguishing characteristics of woman which constitute her peculiar individuality and value, and seeing also with equal clearness that the average man is deficient in, and defective by the want of, those very qualities of which the word "woman" is a sufficiently descriptive label—such a man cannot help eagerly desiring that women may come forward in the world, that their peculiar qualities may be strengthened and intensified, and that they may fill out those deficiencies which so strikingly detract from the perfect manhood of mankind.

To put hindrances in the way of the free exercise of the mental and moral faculties peculiar to women may be very natural and very human, but it is most certainly very stupid. No one will say that the total sum of intellect in the world is at all excessive; still less will it be affirmed that pure morality is too large a quantity, Every man who is not a hopeless fool, as unhappily too many men are, will welcome with enthusiasm both intellect and morality, from whatever source they come. The problem of life is for all men and all women to solve, and to unite in solving. No single man, with his limited allowance of mental and moral faculty, can solve it, nor can any single woman. The whole race of living men and women have unitedly to understand and make clear the mental and moral complexities of their own time. With each succeeding generation the new problems become more difficult; but so also is there a corresponding increase of accumulated knowledge, and a strengthening of the inherited mental powers. If evolution be sound, and Christianity true, this process will continue so long as the human race endures. If the solution in any given age is to be perfect and satisfying, it can only be made so by the uuited efforts of all the best intellects of the time, both male and female.

Now, if these positions can be maintained by a rational dialectic—and it seems to the writer that they are as obvious as they are elementary—we may go so far as to say that the time has arrived when duty demands that women shall claim their full share of the thinking, philosophising, teaching, law-making, and practical working, which have hitherto been almost entirely monopolised by men. It is true that women have been met by many rebuffs, often of a very rude and stupid kind. But therein they have only shared the fate of all pioneers. It is truly wonderful to a philosophic mind what unanimity of speech and action can be evoked from mankind in favour of what is. No matter how irrational, how inconvenient, how injurious, how flagrantly monstrous even a thing may be, if it is actually existent, and can boast of antiquity, however limited, the whole world will rush to its defence and insist upon cause being shown why it should be removed or even assailed. And that conservatism has its decided merits when it is not carried to excess. That natural, and in many respects meritorious, conservatism, women have to fight against. But they are only asked to show cause. No thinking mind condemns them unheard. It is only the dull pedants and the interested blockheads who do that. Have they shown, are they showing, can they show that they possess intellect, moral faculty, purpose, continuity, persistent energy which carries purpose to completion, of such a character as to rank with these endowments in man, or to complement or supplement his? We contend that they can, and we purpose in a short series of papers to justify this contention.

*(To be continued.)*

